# 纳升液相色谱仪器的研究进展

**DOI:** 10.3724/SP.J.1123.2021.06017

**Published:** 2021-10-08

**Authors:** Sandong YANG, Naijie LI, Zhou MA, Tao TANG, Tong LI

**Affiliations:** 1.中国科学院苏州生物医学工程技术研究所, 江苏 苏州 215163; 1. Suzhou Institute of Biomedical Engineering and Technology, Chinese Academy of Sciences, Suzhou 215163, China; 2.大连依利特分析仪器有限公司, 辽宁 大连 116023; 2. Dalian Elite Analytical Instruments Co., Ltd., Dalian 116023, China; 3.依利特(苏州)分析仪器有限公司, 江苏 苏州 215123; 3. Elite Suzhou Analytical Instruments Co., Ltd., Suzhou 215123, China

**Keywords:** 小型化, 纳升液相色谱, 柱外效应, 输液装置, 进样装置, 管路与连接, miniaturization, nano liquid chromatography, extra-column effect, solvent delivery equipment, injection equipment, tubing and connection

## Abstract

小型化是液相色谱分离技术发展的重要趋势之一,包括仪器外形尺寸的小型化、分离材料粒径的小型化以及色谱柱内径的小型化。色谱柱内径的减小能够降低样品和流动相的消耗,具有更高的质量灵敏度,特别适合用于复杂样品体系的分离分析。纳升液相色谱一般是指使用内径小于100 μm的毛细管色谱柱,流速范围在每分钟几十至几百纳升的色谱技术。由于流速很低,色谱柱体积很小,柱外效应显著,因此对色谱仪器系统各个模块的性能以及系统柱外效应的优化提出了较高的要求。纳升液相色谱的输液装置需要能够准确稳定地输送纳升级流速,具有梯度输液模式,且拥有一定的耐压能力,以适应不同规格的色谱柱类型;进样装置需要能够进行准确重复的进样过程,进样体积及进样方式适合毛细管色谱柱,同时不产生明显的柱外效应;检测装置需要具有较高的灵敏度,且具有较小的柱外扩散;管路与连接系统需要稳定、可靠、易操作,并能够最大限度地减小柱外体积,适配纳升级流速。鉴于目前大多数纳升液相色谱系统与质谱检测器联用,因而本文主要从输液装置、进样装置、管路与连接3个方面对相关技术领域的研究论文、技术专利以及仪器厂商的宣传文件等进行了检索与归纳,综述了这些模块的技术路线与研究进展,同时简要介绍光学吸收型检测装置的优化思路与研究进展,并对部分商品化的纳升液相色谱系统进行了对比。

## Abstract (en)

The miniaturization of liquid chromatography equipment is among the most important focus areas in chromatographic technology. It involves the miniaturization of the physical dimensions of the instrument, size of the separation material, and inner diameter of the column. The advantages of a reduced inner diameter of the column have been investigated for several decades, and can be summarized as follows. First, the sample consumption is lower, which is particularly beneficial when a limited amount of sample is available, as is the case with natural products, and in biochemistry and biomedicine. Second, the consumption of the mobile phase is reduced, making the process environmentally friendly and facilitating green chemistry. This allows the addition of more expensive solvent additives, such as chiral additives or isotopic reagents, while maintaining a low analysis cost. Moreover, the degree of band dilution in the column is lower than that with conventional liquid chromatography under the same sample injection conditions. Thus, enhanced mass sensitivity is achieved. Other benefits of a reduced inner diameter of the column include temperature control due to effective heat transfer through the columns and easier coupling to mass detectors, which is particularly advantageous for analyzing complex samples. Typically, the term “nano liquid chromatography” is associated with liquid chromatography, which employs capillary columns of inner diameters less than 100 μm and flow rates in the range of tens to hundreds of nanoliters per minute. Because of the extremely low flow rates and small column volume, the extra-column effect becomes more prominent. Thus, the requirements for every component of liquid chromatographs are augmented toward improving their performance and optimizing the extra-column band broadening of the entire system. The solvent delivery equipment should be able to pump mobile phases accurately and steadily at nanoliter-level flow rates. A gradient mode is required to achieve this, which implies that the lowest flow rate for a single pump unit should reach a few nanoliters per minute. A certain operating pressure is also necessary to employ columns with different inner diameters and particle sizes. A precise and repeatable sample injection procedure is essential for nano liquid chromatography. The injection volume and mode should be suitable for capillary columns, without inducing a significant extra-column effect. A higher-sensitivity detector should be employed, and sample dispersion should be limited. The improved tubing and connection method in nano liquid chromatography should offer stability, reliability, and ease of operation. The extra-column volume should also be restricted to suit nanoliter-level flow rates. Considering that most nano liquid chromatographic instruments have been coupled with a mass detector, this review mainly focused on nanoliter solvent delivery modules, sample injection modules, and tubing and connection modules. By searching and summarizing research articles, technical patents, and brochures of instrument manufacturers, technical routes and research progress on these modules were described in detail. The pump designs can be classified into four types. Pneumatic amplifying pumps have been used in ultra-high-pressure applications. The flow-splitting delivery system, though easy to realize, may lead to a large amount of solvent wastage. Splitless pumps, which are classified based on two main principles, are widely used. Some pumps based on other physical phenomena have been suggested; however, they lacked stability and robustness. Two types of injection modes have been utilized in nano liquid chromatography. The direct nanoliter injection mode typically takes advantage of the groove on the rotor of a switching valve. The trapping injection mode uses trap columns to enable the introduction of large sample volumes. As for the tubing and connection, a few appropriate designs can be acquired from commercial suppliers. The robustness has been improved using some patented technologies. The optimization principles and research progress on optical absorption detection are briefly introduced. Finally, commercial nano liquid chromatographic systems are compared by considering the pumps and injectors.

液相色谱技术经过一个多世纪的发展,已成为应用最广泛的分离分析技术之一。液相色谱原理、分离技术、液相色谱仪器的不断创新,扩展了液相色谱的应用范围,为分析化学家带来更加高效的分离工具。在液相色谱的众多发展方向中,降低色谱柱的内径所带来的优势使其得到了长期的关注与研究^[1,2,3,4,5,6]^。色谱柱内径的降低可减少样品和流动相的消耗,不仅特别适合样品量受限时的分离分析,而且符合绿色化学的发展理念。当色谱柱单位截面积的进样浓度大于常规色谱柱时,样品的稀释效应会小于常规色谱柱,展现出更高的检测灵敏度^[7]^。色谱柱内径的降低也会使色谱柱的温度控制更加容易,降低高压分离带来的温度效应^[8]^。此外,由于分离流速的降低,更易于与质谱仪联用。然而,柱外效应是限制这一发展方向的主要问题,且色谱柱内径越小,柱外效应的影响越显著^[9]^。

纳升液相色谱是伴随着色谱柱内径的不断降低而出现的技术。“纳升”往往指的是流速范围每分钟几十至上百纳升,样品量几纳升至上百纳升,有时也包括检测池体积几纳升至几十纳升,其使用的色谱柱内径一般介于10~100 μm之间^[10]^。目前,纳升液相色谱已成为蛋白质组学^[11]^、代谢组学^[12]^、脂质组学^[13]^等领域的重要研究工具,并且在食品分析^[14]^、药物分析^[15]^、环境污染物分析^[16]^等常规分析领域有所应用。

相较于使用常规流速与常规尺寸色谱柱的液相色谱技术,纳升液相色谱技术发展缓慢,主要是受限于稳定可靠的仪器系统的研发^[17]^。在纳升液相色谱系统中,溶剂输送装置需要产生准确、稳定的纳升级流速,并且能够实现纳升级的梯度输液;进样装置需要能够在不产生明显柱外效应的情况下实现准确重复进样;检测装置需要在纳升级流速下不产生明显的柱外效应,且具有较高的灵敏度;管路及连接也需要进行优化以最大限度地减小色谱峰展宽,适配纳升级的分析流速。对这些模块的研究和改进是提高纳升液相色谱系统的性能,使其能够在更多应用领域发挥作用与优势的关键。

目前,纳升液相色谱系统多与质谱仪串联使用,应用紫外检测器或荧光检测器的相对较少。一些商品化仪器甚至仅将输液泵和自动进样器进行集成,不配置检测器。因此,本文主要从输液装置、进样装置、管路及连接3个方面综述构成纳升液相色谱仪器主要模块的研究进展,对检测装置仅作简要介绍,并对部分商品化的纳升液相色谱系统的技术路线及性能参数进行了对比。

## 1 溶剂输送装置

### 1.1 气动放大泵

气动放大泵是最早应用于纳升液相色谱中的输液系统。在MacNair等^[18,19]^对超高效分离填料的研究中,分别用直径1.5 μm、1.0 μm的颗粒装填到30 μm内径的不同长度毛细管中,由于反压可达100 MPa左右,故使用多级的气动放大装置,以恒压模式进行毛细管色谱柱的装填与评价。虽然这种输液装置能够产生非常大的输液压力,并可利用稳定气源实现稳定地输液,但气动放大泵在往复运动或补充流动相时会产生较大的脉动,且难以通过直接控制输出流速实现梯度输液,限制了其进一步的实际应用。目前,这种模式的输液装置一般在需要极高压力下装填纳升色谱柱时才会有所应用^[20]^。

### 1.2 分流输液装置

分流是实验室研究中最常使用的一种纳升流速输液技术^[21]^,也是部分商品化仪器所采用的技术。分流的原理是利用三通装置将常规输液泵的输出流速进行分流,分流比为两条流路阻力比的倒数。其优势在于结构比较简单,易于实现,利用细内径毛细管调整分离流路和废液流路的阻力比,即可将毫升或微升级流速分流为纳升级流速。对于等度的分离条件来说,尽管分流比很大,但可以将废液流路中的流体回流至溶剂瓶中,节省流动相消耗。然而在梯度分离模式下,由于梯度的混合过程在分流三通之前,无法将废液流路的流动相回收利用,因而流动相浪费较多。尽管分流技术能够简单快速地实现纳升级流速的输出,但由于不同的色谱柱具有不同的反压,因而无法在更换色谱柱或色谱柱状态变化后维持恒定的分流比,分流重复性难以保证。此外,在梯度运行过程中,由于两条流路总体积的不同,二者的流路阻力会随流动相比例的变化而发生非同步变化,从而使分流比和分流流速无法保持恒定。

主动分流调节技术能够缓解系统分流比的改变,如图1所示。图1a^[22]^和图1b^[23]^中的分流装置由固定分流比和可调分流比两部分组成,且分别使用压力传感器和流量传感器来反馈调控可调流路的阻尼。当系统阻力出现变化,使总分流比发生改变时,通过调整可调分流比部分的阻尼,维持总分流比的恒定。图1c^[24]^则是通过电磁比例阀(electro-magnetic proportional valve, EMPV)直接调整分流比,并利用在线流量计的反馈信号控制分流输出流速。上述技术的应用尽管缓解了分流过程中压力变化的影响,但未能解决梯度输液过程中的流动相浪费问题。

**图1:**
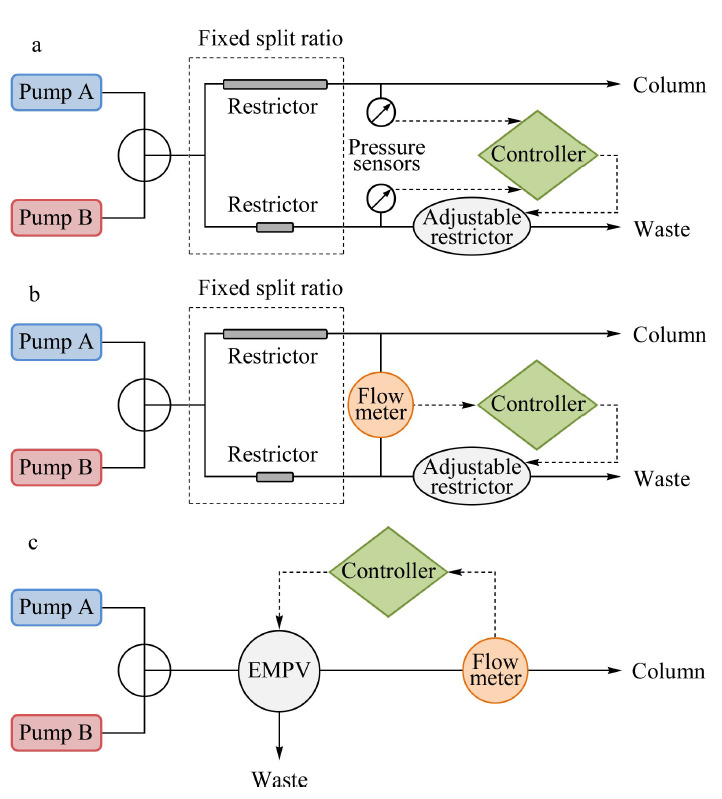
主动分流调节技术示意图

### 1.3 不分流输液装置

不分流的纳升流速输液装置由于能够极大地节省流动相,且流速稳定性较高,是纳升流速输液装置研制与应用的主流。目前主要有两种基本结构,一种是基于双柱塞交替输液的连续输液泵结构^[25,26]^,一种是基于高精度电机驱动的注射泵结构^[27]^,如图2所示。

**图2:**
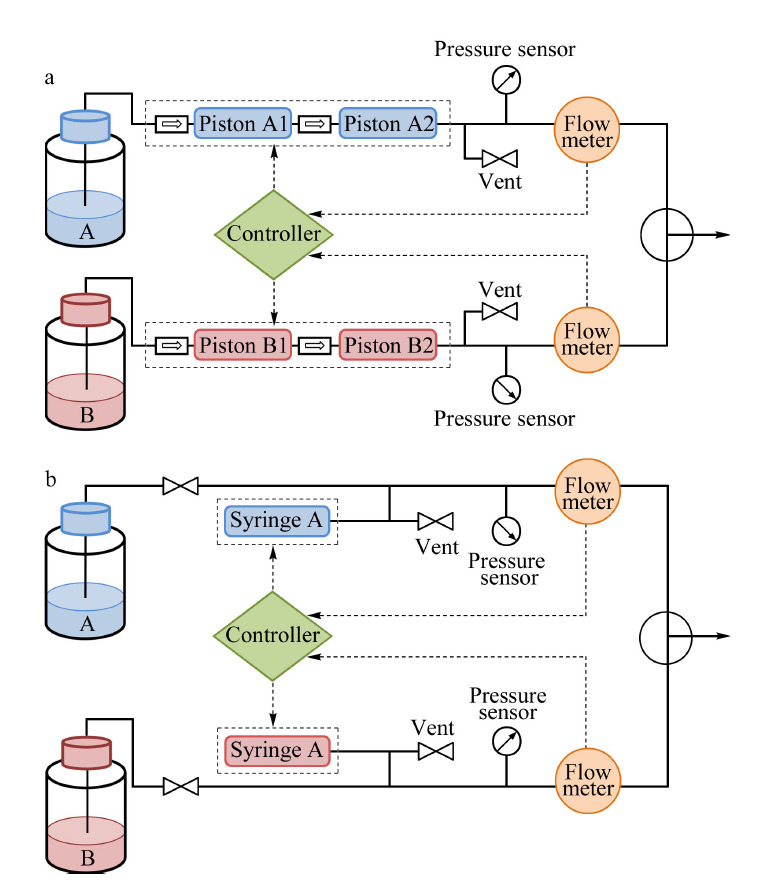
不分流纳升输液装置示意图

连续输液泵的工作原理与常规HPLC或UHPLC的输液泵相近,每种流动相由高精度电机驱动两个柱塞交替输送,根据流路连接以及柱塞的交替方式,又可分为串联与并联结构。与常规输液泵不同的是,由于纳升流速的输出容易受到影响,因此在每种流动相的出口处需设置在线流量传感器,根据实时测量的流速值反馈调控电机的转速,提高纳升流速的准确性和稳定性。

注射泵的工作原理是每种流动相仅由单一柱塞输送,当泵腔内溶剂输送完全后,此次输液过程结束,开始吸液过程并准备下一次输液。这种注射泵的泵头由于只有一个出口,一般利用高压切换阀来选择泵头出口与溶剂瓶还是与系统相通。同时,也同样需要在每种流动相的出口处设置在线流量传感器,反馈调控纳升级的输出流速。这种结构的最大问题是稳定输液时长受到泵腔容积的限制,无法实现长时间连续输液。但由于纳升液相色谱常用的流速为每分钟几百纳升,且分析时间多在1~2 h以内,因此增大泵腔的容积则能够满足大多数的色谱分离需求。Sharma等^[28]^构建了一种便携式纳升梯度液相色谱系统,泵头总容积为74 μL,若输液泵以800 nL/min的流速运行,理论最长运行时间可超过90 min。

目前商品化的纳升液相色谱系统多数采用不分流的纳升流速输液装置,两种结构的优缺点见表1。尽管商品化纳升液相色谱输液泵均利用在线流量传感器反馈调节电机转速,从而提高流速的准确性,但使用时需要注意的是,在线流量传感器只能保证校准流动相的流速准确性。若更换流动相,则需要以更换后的流动相对在线流量传感器进行重新校准。

**表1:** 两种不分流纳升输液装置的优缺点

Pump type	Advantages	Disadvantages
Syringe pump	compact structure, stable during experiment, no check valve in pump head, convenient maintenance	limited delivery time due to pump cavity, inevitable system equilibrating procedure before every analysis
Continuous flow pump	no limit to delivery time, fast equilibration between continuous experiments	complex structure and control algorithm, higher failure rate caused by multiple check valves

在“十二五”国家重大科学仪器设备开发专项的支持下,本团队承担了“多微生物色谱仪及液质联用关键部件的研制”项目的子任务“超高压微纳液相色谱系统的研制”。本团队^[29]^在此项目的研究工作中,研制了一种注射式纳升梯度输液泵,泵腔总容积可达180 μL。该纳升输液泵利用2个高精度直驱电机作为动力源,同时使用刚性的柱塞连接装置,确保电机的运动精度可直接传递至输液柱塞。在其流路结构设计上取消了传统的单向阀模块,采用耐超高压的二位十通阀作为输液吸液状态转换的接口,解决了纳升级的流体微渗问题,使输液泵耐压超过100 MPa。流速与梯度输液测试结果表明,在500 nL/min条件下的流速准确性、稳定性以及梯度偏差均低于1%。以BSA酶解液及Hela细胞蛋白质酶解液进行评价,评价结果与进口仪器相当,可应用于蛋白质组学的分析研究中。

### 1.4 基于其他物理现象的输液装置

由于纳升流速比较微小,一些能够产生轻微形变或位移的物理现象,也可以用于驱动液体流动,实现无分流的纳升流速输液。

电渗泵是研究较多的一种微流量泵,其原理是利用载流的电渗现象驱动流动相运动^[30]^,载流微通道可为空管^[31]^、填充柱^[32]^、整体柱^[33]^、多孔膜^[34]^等形式,如图3所示。通过控制驱动电压的极性,控制载液的流动方向,配合阀结构实现输液和吸液。Zhou等^[35]^利用整体柱作为载流微通道,构建了一种二元梯度电渗泵,最大输出流速为490 nL/min。尽管电渗泵能够实现纳升流速的输送,装置成本较低,但由于最大输液压力一般不超过40 MPa,且输液的流速与色谱柱背压有关,是一种恒压泵,因此应用受到一定限制。

**图3:**
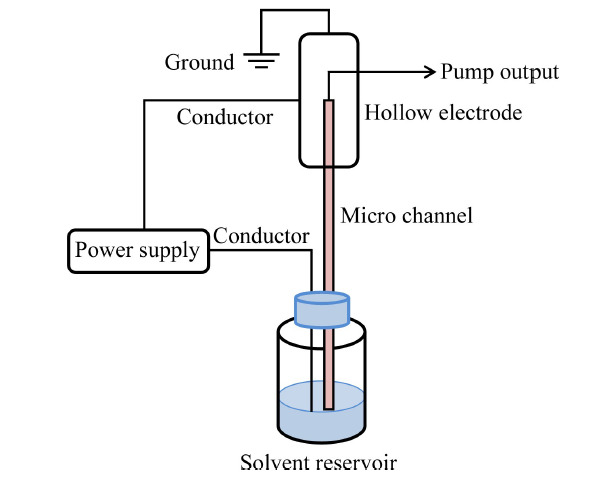
电渗泵结构示意图

磁致伸缩泵^[36]^是通过外加磁场改变磁致伸缩体的形状,驱动柱塞或隔膜的前后运动,再配合阀结构,实现输液和吸液。磁致伸缩体的形变量较小,通过设计隔膜或柱塞与磁致伸缩体的直径比,调节输出的流速范围。磁致伸缩泵的优点是机械结构简单,流速下限低。但目前尚无用该泵进行梯度输液的研究,且磁致伸缩体的形变控制比较复杂,离实际应用还有一段距离。

热膨胀泵^[37]^是一种基于液体受热膨胀的物理现象驱动流动相流动的输液泵,由于液体受热膨胀量较小,因而能够输出纳升级的流速。张祥民等^[38]^研制了一种可实现梯度输液的热膨胀泵,在500 nL/min的流速条件下,流速重复性的RSD为4%,色谱分离保留时间的重复性RSD为2%。

本团队^[39]^基于物质发生固液相变时能够产生体积变化,研制了一种相变泵。若物质发生固液相变时体积增大,则可通过对固态物质加热使其发生相变,利用体积膨胀的作用,驱动流动相。实验测试这种相变泵的最低输出流速可低至100 nL/min,输液压力可达80 MPa。但由于均匀、精确且迅速的控温过程难以实现,流速的稳定性并不理想。

基于不同原理实现纳升流速的输液装置汇总见表2。

**表2:** 纳升流速输液装置汇总

Type	Driving principle	Flow rate range/ (nL/min)	Maximum pressure/ MPa	Continuous delivery or not	References
Pneumatic amplifying pump	pneumatic with multiple amplification	12-1.7×10^6^	900	no	[18,19]
Active flow splitting systems	split ratio is inversely proportional to resistance ratio	50-2.5×10^6^	40	yes	[22-24]
Continuous flow pump	high precision motors driving double pistons in	20-1×10^5^	100	yes	[25,26]
	series/parallel				
Syringe pump	high precision motor driving single piston	20-2×10^3^	120	no	[27-29]
Electroosmotic pump	electroosmotic phenomenon	0-5×10^4^	40	no	[34,35]
Magnetostriction pump	magnetostrictive effect	-	130 kN	no	[36]
Thermal expansion pump	thermal expansion of liquid	10-5×10^4^	10	no	[37,38]
Phase transition pump	volume change during phase transition of liquid or solid	minimum 100	80	no	[39]

-: not provided.

## 2 进样装置

样品的进样过程不仅影响定量结果的重现性和准确性,同时也会因为柱外效应,对色谱分离效果产生影响^[40,41,42]^。特别是对于纳升液相色谱,随着色谱柱尺寸的明显减小,进样量也需要随之大幅降低,以避免发生质量超载或体积超载^[43]^。根据估算^[10]^,纳升色谱柱的进样体积一般在几纳升至几百纳升之间。因此,适合纳升液相色谱系统的进样方式主要有纳升体积进样和捕集进样两种模式。

### 2.1 纳升体积进样

常规HPLC的进样体积一般为几微升至几百微升,通过切换阀上的外置定量环即可完成进样过程,且不会对色谱分离造成明显影响。但对于纳升液相色谱系统来说,常规外置定量环的体积过大,若使用细内径的管路制作纳升体积的定量环,则定量环的反压过大,难以使样品顺利进入。因此外置定量环的进样模式不适合纳升液相色谱。

在20世纪末,由于机械加工水平的限制,出现了一些纳升体积的进样方式,如中心切割^[44]^、分流进样^[45]^、柱上聚焦^[46]^等。随着机械加工精度的提升,目前已经可以加工出内置纳升体积定量环的进样阀。如VICI公司的C4N-4004系列商品化进样阀可实现4、10、20 nL 3种纳升体积进样,测试表明使用4 nL进样阀的样品峰面积相对标准偏差优于1%^[47]^。Sharma等^[48]^研制了一种便携式纳升液相色谱系统,通过对切换阀内的转子进行设计和精密加工,使切换阀不仅具有60 nL体积的进样功能,同时还具有纳升注射泵流动相输出与吸入的切换功能,极大地缩小了系统尺寸,如图4所示。内置定量环式进样阀的主要问题是定量环体积固定,若需进样其他体积,一般需要更换转子,甚至更换整个进样阀,使用不方便。Gerhardt等^[49]^发明了一种具有可变体积内置定量环的进样阀,该进样阀转子的转动角度连续可变,通过在转子上加工较长的样品和流动相通道,使转子上的内置定量环区域大小可调,从而控制内置定量环的进样量,如图5所示。

**图4:**
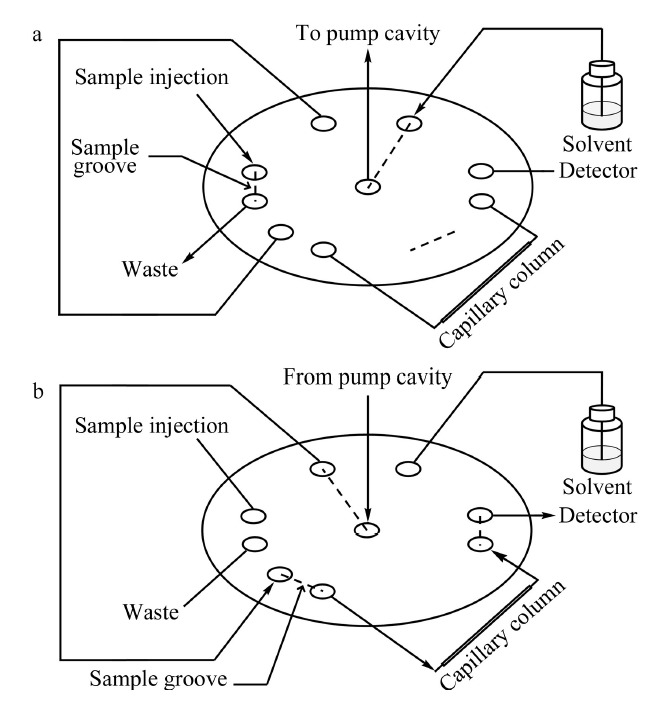
集成式进样阀装置示意图

**图5:**
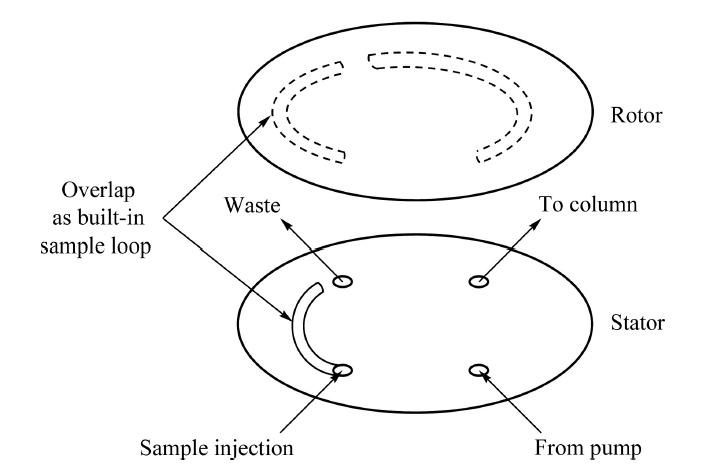
可变体积进样阀示意图

定时进样模式也可以用于实现纳升体积进样,当进样阀上的定量环中引入样品后,通过控制进样阀处于“injection”位置的时间来控制进样体积,进样体积为该时间与流速的乘积。这种进样模式的样品扩散较低,可降低色谱峰拖尾。Prüß等^[50]^对比了该进样模式与部分定量环进样模式下样品分离时的色谱峰展宽,在谱带展宽方面,定时进样模式的结果更好。

### 2.2 捕集进样

虽然通过理论计算,适合纳升液相色谱的进样体积应该在纳升级,但直接纳升体积进样方式不仅可能受到进样装置的限制,对样品的浓度也有一定的要求。在生化分析领域,样品的浓度往往很低,纳升体积进样并不适用。捕集进样方式能够以微升级体积进样,并在捕集柱上对样品进行浓缩,也可以在捕集柱上实现除盐、调整pH等操作,是纳升液相色谱应用最多的一种进样方式^[51]^。其原理是利用低洗脱能力的流动相将较大体积的样品在捕集柱的柱头上进行样品的富集,之后通过阀切换,以较高洗脱能力的流动相将样品洗脱至分析柱上进行后续分离。由于捕集柱的富集浓缩作用,样品谱带展宽较小。Leonhardt等^[52]^研究发现,使用较短的捕集柱、较高的捕集流速更有利于大体积样品的在线浓缩。

捕集进样主要有两种流路连接方式,如图6所示。一种是样品通过进样阀进样后,由纳升分离泵驱动样品捕集,捕集柱后连接排空切换阀。捕集时,排空阀打开,捕集后的流动相从排空阀排出;分析时,排空阀关闭,捕集柱与分析柱相连,进行样品的洗脱^[53]^。另一种是样品通过进样阀进样后,由上样泵驱动样品捕集,捕集后进行阀切换,使捕集柱与纳升分离泵、分析柱相连,进行样品的洗脱^[54]^。

**图6:**
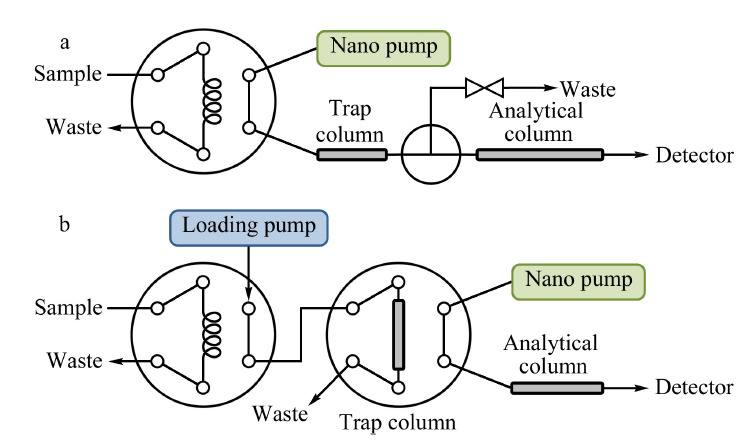
捕集进样方式的示意图

两种流路结构相比,前者可节省一个上样泵和一个六通切换阀,但捕集过程与分离过程的系统压力变化较大,对系统的快速升压提出更高的要求;后者的系统压力稳定性更好,但装置成本较高。在使用捕集进样方式时,也需要注意低保留组分在捕集柱上的损失问题,以及引入捕集柱所引起的梯度延迟问题。

除了上述利用低洗脱能力的流动相对样品进行捕集进样的方法以外,也可以利用温度的控制实现大体积样品的捕集进样。由于纳升色谱柱的直径很小,可以实现快速的温度变化^[55]^,从而实现温度辅助柱上样品聚焦(temperature-assisted on-column solute focusing, TASF)^[56]^。样品进样后,对色谱柱前端的7 mm长度进行制冷,使其温度在30~45 s内降低至5 ℃,实现样品的捕集。之后再对这段色谱柱进行快速升温至60 ℃,释放捕集的样品进行后续分离分析。通过这种方式,能够显著降低色谱峰的展宽。将TASF进样方式与基于低洗脱能力流动相的捕集进样方式结合,可以进一步提高捕集效果,增大进样体积^[57]^。

表3对比了上述几种纳升LC的进样方式。

**表3:** 纳升液相色谱进样方式对比

Injection mode	Advantages	Disadvantages	References
Built-in sample loop	simple, robust, no sample loss	limited injection volume and peak capacity, no protection of the separation column	[47,48,58,59]
Variable-volume injection valve	robust, no sample loss, variable injection volume	higher machining precision, no protection of the separation column	[49]
Timed injection	variable injection volume, simple	precision affected by time and flow rate, no protection of the separation column	[50]
Trapped on a vented column	sample wash, greater injection volume, small dead volumes, protection of the separation column	interruption of the flow, pressure and spray, limited robustness, possible sample loss on the trap column	[53,60]
Trapped by column switching	sample wash, greater injection volume, robust, protection of the separation column	possible sample loss on the trap column, more modules	[54,61,62]
TASF	greater injection volume, small dead volumes, lower dispersion	repeatability affected by temperature control, baseline disturbance	[56,57,63]

TASF: temperature-assisted on-column solute focusing.

## 3 管路及连接

管路与连接方式是产生柱外效应的重要部分^[64,65,66]^,不适合的管路与不正确的连接方式会显著影响纳升液相色谱的分离结果,这也是很多纳升液相色谱系统得不到理想实验结果的主要原因。

系统内接触流体的管路材质常用不锈钢、PEEK(聚醚醚酮,poly-ether-ether-ketone)、石英毛细管、钛合金、MP35N(一种镍铬钴合金)等。不锈钢管路机械强度高,多用于纳升输液泵内部各组件间的连接,内径尺寸多为0.127 mm (0.005英寸)或0.178 mm (0.007英寸),但由于其对生物样品的兼容性不好,进样后的各连接管路几乎不使用不锈钢材质。此外,由于不锈钢还可能释放金属离子进入流动相中,影响色谱柱或样品,在一些输液泵的设计中也使用其他材质替代不锈钢管路。在连接纳升液相色谱系统间各个模块时,PEEK与石英毛细管是最常用的两种管路材料,其生物兼容性较好,且较不锈钢管路更易弯曲,方便使用。PEEK管路外径一般为0.794 mm (1/32英寸),内径一般不超过0.127 mm (0.005英寸),但PEEK材质不耐受四氢呋喃、二氯甲烷、二甲亚砜等有机试剂。石英毛细管外径一般为360 μm,内径一般在20~100 μm之间,但石英毛细管易碎,增加了系统的不稳定性。一种解决方案(PEEKsil)是将PEEK材质包裹在石英毛细管外,使其兼具了二者的优点。因为常规的切割PEEK或石英毛细管的方式会使其受损,一般只使用预切长度。尽管连接管路的内径越小,柱外效应越小,且一些厂家有更细内径的石英毛细管产品,如10 μm或小于1 μm^[67]^,但连接管路的阻力会随着内径的降低而显著升高,甚至超过整个系统的耐压^[42]^,因此对于每分钟几百纳升的流速条件,20 μm通常是纳升液相色谱连接管路内径的下限。

纳升液相色谱的连接接头主要有两种形式,一种是刃环密封的连接方式,一种是端面密封的连接方式。刃环密封的连接方式是从常规HPLC系统延续而来的,通过刃环外表面与锥孔内表面、刃环内表面与管外壁的两级密封方式,实现流路的连接。由于目前纳升液相色谱系统的连接孔多为适配外径1.588 mm (1/16英寸)的管路、10-32螺纹的螺丝,若使用外径0.794 mm (1/32英寸) PEEK管或外径360 μm的石英毛细管,则需要使用套管结构来辅助连接,如图7a所示。刃环密封的连接方式最主要的问题是容易产生死体积^[68]^。理想的连接方式是刃环与管路前端的距离刚好适合连接孔且管路前端与连接孔底端紧密相连。但实际连接时,管路前端与连接孔底端的紧密程度靠螺丝旋进的深度调整,深度过小,端面密封不好,深度过大,容易使刃环对管路造成挤压或损坏,不易控制,对操作者的经验要求较高。端面密封的连接方式是通过特殊设计的管路前端及接头结构,使得螺丝在旋入连接孔时,直接将管路前端挤压在连接孔的底端,实现端面密封。密封的程度可以通过旋转螺丝的扭矩进行控制,不会对管路造成损伤,如图7b所示。目前Thermo Fisher Scientific公司的Viper系列^[69,70]^和IDEX公司的MarvelX系列^[71]^均采用这种设计。Stankovich等^[65]^测试发现,这种连接方式可以显著降低色谱峰展宽。由于这种连接结构需要具有特殊设计的管路前端,因此均为预切长度或定制长度的管路,无法根据实际需求随时调整。

**图7:**
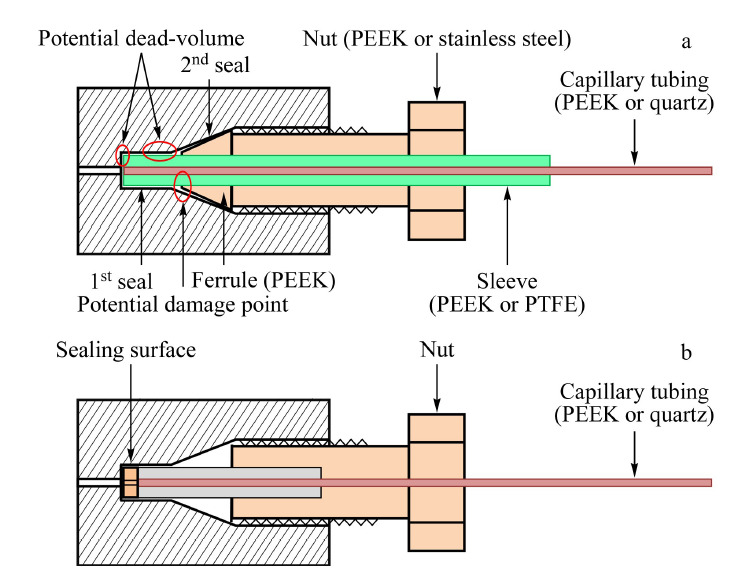
连接方式

## 4 检测装置

纳升液相色谱系统可使用的检测器类型主要有光学吸收型检测器^[72]^、荧光检测器^[73]^、质谱检测器^[74]^等。其中,质谱检测器应用最广泛,但与光学吸收型检测器相比,有关质谱检测器对柱外效应贡献的研究相对较少^[75,76]^,色谱柱出口与质谱检测器入口之间的连接管路是其柱外效应的重要组成部分^[77]^。对于使用电喷雾离子源的质谱检测器(electrospray ionization-mass spectrometer, ESI-MS),将毛细管柱末端制成尖锐的锥形,直接用于电喷雾离子源中,避免使用额外的连接管路,可以有效避免柱后展宽^[78,79]^。

光学吸收型检测器是基于样品流过流通池时吸收光强的多少进行定量,流通池的光程和池体积分别影响检测的灵敏度和样品在池内的扩散^[80]^。由于同样使用毛细管色谱柱,毛细管电泳系统、毛细管电色谱系统所使用的流通池设计思路也可用于纳升液相色谱系统中^[81]^。与毛细管电泳系统、毛细管电色谱系统相似,对于纳升液相色谱系统来说,柱外效应及样品的扩散对于分析检测结果的影响更大,因此通常采取减小光程,牺牲灵敏度的流通池设计策略。柱上检测是这一类系统中最常用的流通池设计,通过去掉毛细管色谱柱末端的聚合物保护层,使光线直接穿过毛细管壁^[82]^或毛细管柱^[83]^进行检测。这种检测方式的灵敏度很低,流通池光程仅与毛细管内径相当。为了提高检测灵敏度,可通过在毛细管中鼓泡^[84,85]^、毛细管径向多重反射^[86]^、毛细管轴向Z型池^[87,88]^等方式,在不显著增加流通池体积的前提下(<100 nL),增大光程。更小的池体积结构也往往意味着更低的通光量,会增加噪声对检测灵敏度的影响。对于基于轴向照射设计的流通池,为了提高光通量,可以使用Teflon AF作为池内壁材料,利用其折射率低于液相色谱常用流动相的特性,实现液芯波导(liquid-core waveguide, LCW)全反射技术,可减小流通池内的光能损失,降低噪声水平,提高信噪比^[89,90,91,92]^。

## 5 商品化纳升液相色谱系统

商品化纳升液相色谱系统目前几乎全部为国外公司的产品,各产品的主要差异在于输液装置的设计及指标参数上。

最早的商品化纳升液相色谱是由荷兰LC Packings公司(后被Dionex收购,目前属于Thermo Fisher Scientific公司)在1998年推出的UltiMate NanoLC系统。该系统的输液装置基于分流的原理,利用预设分流比的流速选择模块进行流速范围的配置(与图1b原理相近)。根据所使用的流速选择模块的不同,流速范围有50 nL/min~1 μL/min、500 nL/min~10 μL/min、10~160 μL/min 3种可选。该系列的最新产品UltiMate 3000 RSLCnano使用双柱塞串联连续输液模式(与图2a原理相近),可实现无分流的纳升输液,流速范围20 nL/min~50 μL/min,耐压可达80 MPa。该输液装置内同时集成了一个三元低压梯度微升泵,可用于样品的捕集过程以及二维色谱,扩展了应用范围。EASY nLC系列纳升液相色谱系统目前也是Thermo Fisher Scientific公司的产品(原属于丹麦Proxeon公司),该系统将输液泵和自动进样器集成在一台仪器内,极大地减小了仪器所占的空间。输液装置使用无分流的双注射泵配置(与图2b原理相近),单泵的泵腔容积为140 μL,其最新型号EASY nLC 1200的流速范围是20~2000 nL/min,耐压可达120 MPa。该产品也多作为OEM(original equipment manufacturer)出现在其他公司的纳升液相色谱-质谱联用系统中,如Bruker和Varian。

Eksigent公司(现属于AB Sciex公司)的纳升液相色谱产品是NanoLC系列。该产品的输液装置同样配置了无分流的双注射泵,且使用一种微流体流速控制技术(microfluidic flow control, MFC)对输出流速进行控制。最新的Ekspert nanoLC 400可通过更换流速模块,实现3个流速范围的选择,整体流速范围为100 nL/min~50 μL/min,耐压69 MPa。

Waters公司的Acquity M-Class系统是由nanoAcquity系统升级而来的纳升液相色谱系统。该系统的输液装置使用二元双柱塞往复连续输液泵,通过流量传感器的闭环反馈控制,可实现200 nL/min~6 μL/min的输出流速,开环控制模式最大可实现100 μL/min的流速。系统的最大耐压超过100 MPa,可用于快速分析。Acquity M-Class系统中除了具有纳升输液装置和定量环式的进样装置外,还具有可用于样品捕集的辅助溶剂输送装置、捕集阀管理装置,以及最大可容纳7296个样品的样品管理器。

Agilent公司的纳升液相色谱系统始于1100系列,其输液装置由双柱塞串联泵系统构成,在流动相混合器之后依次连接电磁比例阀和流量传感器(与图1c原理相近),纳升流速的控制由电子流速控制(electronic flow control, EFC)系统实现。由于该输液装置采用分流原理且分流发生在流动相混合之后,因此流动相的消耗量较大。该系列的升级产品,1200 Infinity系列的纳升输液装置依然采用相似的工作原理,虽然流速下限从1100系列的1 μL/min降低至100 nL/min,但依然存在流动相消耗量较大的问题。该输液装置可以通过更换硬件,实现毫升工作模式的切换。

岛津公司nano Prominence纳升液相色谱系统的输液装置也是采用了分流的设计原理,但其分流过程发生在梯度混合之前,因而也可以节省流动相的消耗。通过回流流速控制(reflux flow control, RFC)系统,利用纳升流量传感器监控分流后每种流动相的输出流速,反馈调控电机转速,使两种流动相以纳升流速进行梯度混合。该输液系统可以在40 MPa的范围内实现1 nL/min~5 μL/min的输出流速。

表4对部分商品化纳升液相色谱系统的指标进行了对比。

**表4:** 部分商品化纳升液相色谱系统的指标参数

Manufacturer	Model	Pump					Autosampler	
		Flow rate range/ (μL/min)	maximum pressure/ MPa	Flow precision (RSD/%)	Delay volume/ nL		Lowest injection volume/μL	Injection repeatability (RSD)
Thermo Fisher	Ultimate 3000 RSLCnano	0.02-50	80	0.2 (300 nL/min)	25		0.01	0.4%(full loop, 1 μL)
Scientific								
Thermo Fisher	EASY-nano LC 1200	0.02-2	120	0.4	1000		0.1	0.2%(pick-up, 5 μL),
Scientific								3.0%(pick-up, 0.1 μL)
AB Sciex	Ekspert nanoLC 400	0.1-50	69	0.35 (500 nL/min)	25		0.1	0.5%(full loop ),
								1%(pick-up, >1 μL)
Waters	ACQUITY UPLC M-Class	0.2-100	100	-	1000		0.1	1%(0.2-1.9 μL),
								0.5%(2-10 μL)
Agilent	1200 Infinity nano	0.01-4	40	0.7	300		-	-
Shimadzu	Nano Prominence	0.001-5	40	-	-		-	-

-: not provided.

## 6 结论

以更少的样品消耗、更短的分析时间和更高的灵敏度,分析更加复杂的样品,是环境、药物、组学等研究领域分析工作者的不断追求。纳升液相色谱使用极细内径的色谱柱且易于与质谱仪联用,已得到越来越广泛的应用。性能良好且稳定重复的仪器系统是获得可靠结果的基本要素之一。然而从纳升液相色谱的出现发展至今,尽管有多种输液方式、进样方式、连接方式的出现,纳升液相色谱依然主要用于一些精密的研究应用中,无法与常规高效液相色谱的应用范围相比。这主要是由于一些纳升液相色谱仪器的设计以及使用方面的问题依然需要进一步提升或解决,如纳升级别的液体微渗、流速稳定性的保证及故障诊断、细内径管路或色谱柱的堵塞、系统柱外效应的控制、易用且可靠的管路连接方式等等。商品化的纳升液相色谱仪器解决了部分上述问题并进行了一些结构优化,但用户需要面对其高昂的价格及维护成本。由于降低色谱柱内径是液相色谱发展的一个重要趋势,因而更低流速的稳定溶剂输送技术、更低柱外效应的系统设计、更高稳定性的仪器集成依然是纳升液相色谱的主要研究方向。
